# RNA-Seq transcriptomic analysis with Bag2D software identifies key pathways enhancing lipid yield in a high lipid-producing mutant of the non-model green alga *Dunaliella tertiolecta*

**DOI:** 10.1186/s13068-015-0382-0

**Published:** 2015-11-25

**Authors:** Lina Yao, Tin Wee Tan, Yi-Kai Ng, Kenneth Hon Kim Ban, Hui Shen, Huixin Lin, Yuan Kun Lee

**Affiliations:** Department of Microbiology and Immunology, Yong Loo Lin School of Medicine, National University of Singapore, Singapore, Singapore; Department of Biochemistry, Yong Loo Lin School of Medicine, National University of Singapore, Singapore, Singapore; Institute of Molecular and Cell Biology, A*STAR, Singapore, Singapore

**Keywords:** *Dunaliella tertiolecta*, Next-generation sequencing, RNA-Seq, Random insertional mutant, Lipid metabolism

## Abstract

**Background:**

For many years, increasing demands for fossil fuels have met with limited supply. As a potential substitute and renewable source of biofuel feedstock, microalgae have received significant attention. However, few of the current algal species produce high lipid yields to be commercially viable. To discover more high yielding strains, next-generation sequencing technology is used to elucidate lipid synthetic pathways and energy metabolism involved in lipid yield. When subjected to manipulation by genetic and metabolic engineering, enhancement of such pathways may further enhance lipid yield.

**Results:**

In this study, transcriptome profiling of a random insertional mutant with enhanced lipid production generated from a non-model marine microalga *Dunaliella tertiolecta* is presented. D9 mutant has a lipid yield that is 2- to 4-fold higher than that of wild type. Using novel Bag2D-workflow scripts developed and reported here, the non-redundant transcripts from *de novo* assembly were annotated based on the best hits in five model microalgae, namely *Chlamydomonas reinhardtii*, *Coccomyxa subellipsoidea* C-169, *Ostreococcus lucimarinus*, *Volvox carteri*, *Chlorella variabilis* NC64A and a high plant species *Arabidopsis thaliana*. The assembled contigs (~181 Mb) includes 481,381 contigs, covering 10,185 genes. Pathway analysis showed that a pathway from inositol phosphate metabolism to fatty acid biosynthesis is the most significantly correlated with higher lipid yield in this mutant.

**Conclusions:**

Herein, we described a pipeline to analyze RNA-Seq data without pre-existing transcriptomic information. The draft transcriptome of *D. tertiolecta* was constructed and annotated, which offered useful information for characterizing high lipid-producing mutants. *D. tertiolecta* mutant was generated with an enhanced photosynthetic efficiency and lipid production. RNA-Seq data of the mutant and wild type were compared, providing biological insights into the expression patterns of contigs associated with energy metabolism and carbon flow pathways. Comparison of *D. tertiolecta* genes with homologs of five other green algae and a model high plant species can facilitate the annotation of *D. tertiolecta* and lead to a more complete annotation of its sequence database, thus laying the groundwork for optimization of lipid production pathways based on genetic manipulation.

**Electronic supplementary material:**

The online version of this article (doi:10.1186/s13068-015-0382-0) contains supplementary material, which is available to authorized users.

## Background

After the oil crises of the 1970s and 1980s, much of the debate about world oil markets centered on the limitations of supply [1]. Alternative fuels become highly demanding. The changes in the overall fuel market environment have led to focus on reassessing long-term trends in liquid fuels markets, such as natural gas plant liquids, biofuels, gas-to-liquids, coal-to-liquids, kerogen (i.e., oil shale) and refinery gain [1]. As an alternative and renewable source of lipid-rich biomass feedstock for biofuels, microalgae have been explored and received considerable attention in the recent years [2]. These microorganisms are able to use the solar energy to combine water with carbon dioxide which is the main component of greenhouse gas emissions to produce biomass [3], which convert sunlight into chemical energy in the form of reduced carbon molecules (carbohydrates, oils/fats). Oils or triacylglycerols (TAGs) can be used directly or by a simple chemical conversion to fatty acid methyl esters as biodiesels [4].

Over the past few decades, several thousand algae and cyanobacteria species have been screened for high lipid content, of which several hundred oleaginous species have been isolated and characterized under laboratory and/or outdoor culture conditions [2]. Among them, *Dunaliella tertiolecta* is considered one of the most promising species. *D. tertiolecta* is a flagellated unicellular marine microalga that belongs to the Chlorophyta phylum. The rational for selecting *D. tertiolecta* in this study lies in its ability to produce large quantities of lipids (up to 67 % of organism dry weight), a high tolerance to salt, temperature and light, rapid growth rate in hyper saline environments which eliminate contaminations from the pure cultures, utilize inorganic nutrients present in saltwater, wastewater or brackish water along with sunlight to produce biomass using CO_2_ as a carbon source through photosynthesis, and it lacks a rigid cell wall which eases product extraction and genetic manipulation [5–10].

Genetic manipulation is a common strategy for enhancement of lipid overproduction in microalgae to channel metabolites to lipid biosynthesis by overexpressing one or more key enzymes in microalgal strains [11]. The understanding of pathways and crucial enzymes is essential to modify microalgal strains. To perform gene manipulation in microalgal strains, their genome information is necessary. Currently, few complete genome sequences of microalgae are available, such as green microalgae *Chlamydomonas reinhardtii*, *Coccomyxa subellipsoidea* C-169, *Ostreococcus lucimarinus*, *Volvox carteri* and *Chlorella variabilis* NC64A [12–16]. However, few of these algae are ideal producers of lipids, and, as such, extensive bioinformatics studies and genetic modifications on other species are needed. Enlarged data analytical capability and improved downstream processing in the NGS technology have been developed in recent years [9, 17, 18], such as the studies to identify and construct lipid and starch biosynthesis and catabolism pathways in the microalga *D. tertiolecta* [9], which applied NGS-based transcriptomics to species without reference genome sequences. Although Hamid et al. [9] provided a good approach to investigating into the transcriptome and annotating partial transcripts, the incomplete nuclear and chloroplast genome sequences of *D. tertiolecta* limited the global transcriptomics studies on RNA-Seq data.

In this study, a *D. tertiolecta* mutant D9 with enhanced lipid production was generated. An in-house program was developed using BLASTX algorithms by comparing with five green algal lineages and one high plant species to construct the draft transcriptomic database of *D. tertiolecta* for RNA-Seq analysis and further target gene manipulation. RNA-Seq analysis elucidated the regulation of lipid synthetic pathways in the D9 mutant.

## Results and discussions

### Mutant selection and physiological characterization

**Fig. 1:**
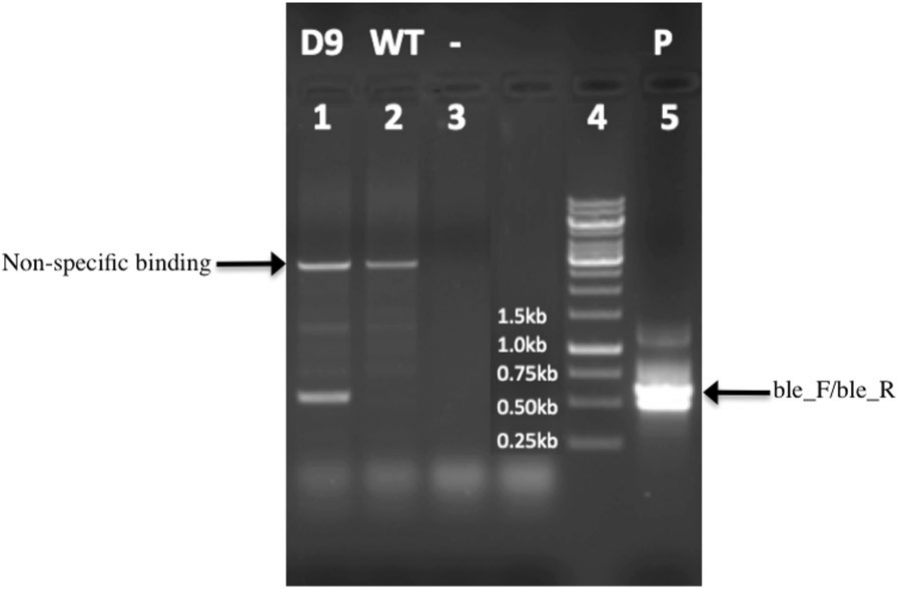
Genotyping PCR results of D9 mutant and WT *D.* *tertiolecta.* The template for *lanes* *1*, *2*, *3*, *4*, and *5*: D9 mutant, wild type, negative control (without template), 1 kb ladder, positive control (using the reconstructed plasmid as the template), respectively. The primer pair for *lanes* *1*, *2*, *3*, and *5*: ble_F + ble_R (the predicted size is 503 bp). The* two arrows* indicate a non-specific binding band and the target bleomycin band. ble_F: AAGCTGACCAGCGCCGTTC, ble_R: CCACGAAGTGCACGCAGTT

Mutants of *D. tertiolecta* subjected to random insertional mutagenesis were generated by transformation using pGreenII0000 plasmid with a bleomycin selection cassette. Zeocin-resistant transformants were screened on 0.08 M ATCC medium agar plates with zeocin. About 30 mutants resistant to zeocin were selected. One transformant with constantly enhanced lipid production was selected and named D9 for further characterization. The bleomycin transgene was detected through genotyping PCR (Fig. 1).

**Fig. 2:**
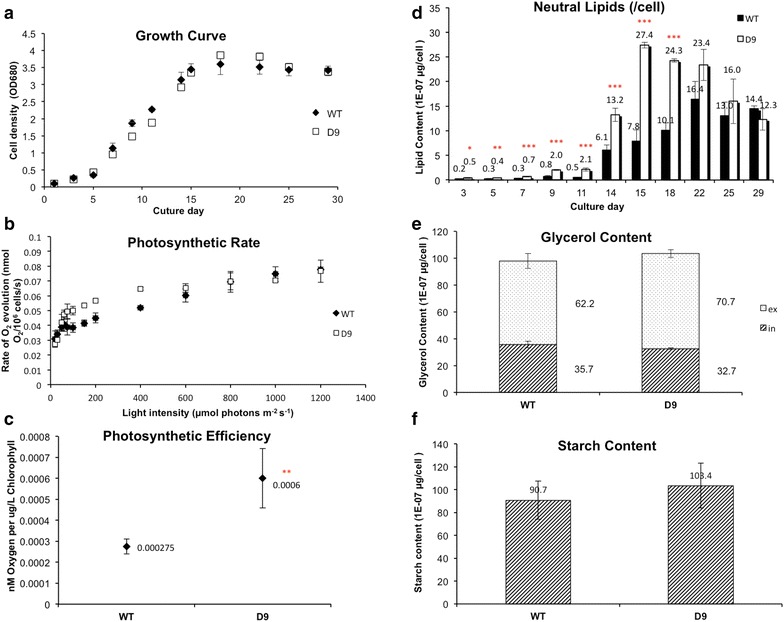
Physiological performance of D9 mutant and WT *D. tertiolecta.* **a** Growth curve of D9 mutant and WT *D. tertiolecta*; **b** photosynthetic rate and maximum photochemical capacity of D9 mutant and WT *D. tertiolecta*; **c** Photosynthetic efficiency of D9 mutant and WT *D. tertiolecta*; **d**, **e**, **f** Detection of carbon fixation parameters, namely neutral lipid content, glycerol content, and starch content. *Asterisk* indicate the statistically significant difference between D9 mutant and WT *D. tertiolecta* after two-tailed T test (*0.01 < p value ≤ 0.05; **0.001 < p value ≤ 0.01; ***p value ≤0.001)

Growth kinetics of the D9 and wild-type *D. tertiolecta* cells grown at 0.5 M ATCC medium were examined and shown in Fig. 2a. In comparison with the wild type, D9 shared the similar growth kinetics. Neutral lipids in D9 was examined and compared with that in the wild type. As shown in Fig. 2d, the D9 mutant produced neutral lipids at the late exponential and early stationary phases at about 2- to 4-fold of that in the wild type, indicating that some carbon flow channeling may be occurring. As an attempt to use a rapid fatty acid detection protocol, we also compared the quantification results obtained from the GC–MS analysis with those obtained from Nile red assays. The Nile red data showed a good correlation with the GC–MS data (R^2^ = 0.86, Additional file 1), indicating such an assay could potentially be used as a high-throughput screen for identifying the next generation of fatty acid-overproducing mutant strains, which was also tested and suggested by Peng Xu [19].

Photosynthetic activities (including rates of photosynthesis, photosynthetic efficiency and the maximum photochemical capacity) of the D9 and wild-type *D. tertiolecta* cells were investigated. D9 mutant in general showed increased photosynthetic performance (Fig. 2b, c), with around twofold increase in photosynthetic efficiency compared to that of wild-type *D. tertiolecta.*

The glycerol production and starch content of D9 and wild-type cells at stationary phase were investigated. Regardless of the neutral lipids production and photosynthetic performance, glycerol production and starch contents in the two genotypes were comparable, as shown in Fig. 2e, f. Lipids and starch are known as endogenous carbon storage compounds [20], and glycerol is the osmo-regulator for *Dunaliella* [21]. The enhanced photosynthetic efficiency resulted in a carbon flux to lipid synthesis and accumulation, rather than starch or glycerol production.

### Pre-analysis and de novo assembly of sequenced data

**Fig. 3:**
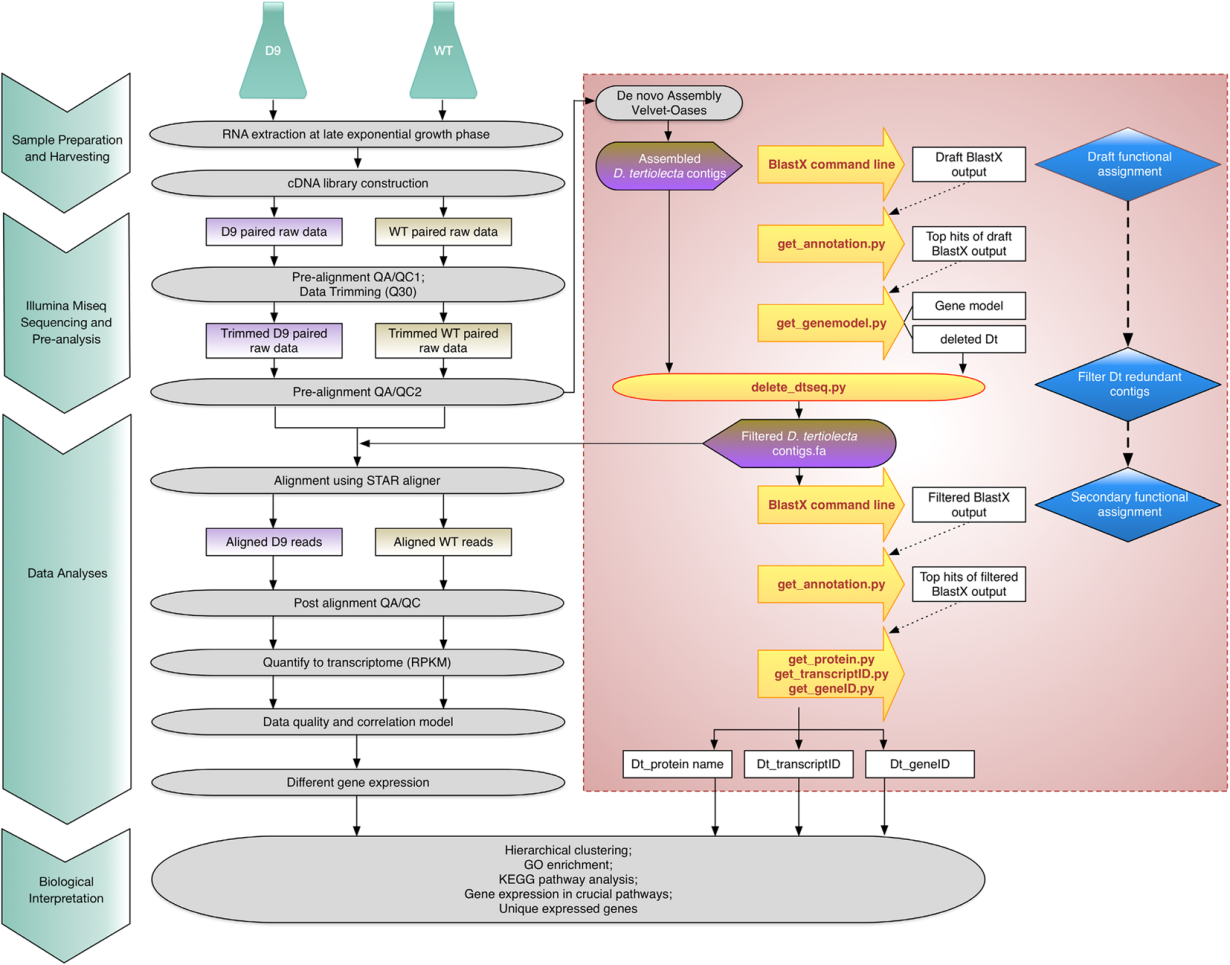
RNA-Seq data analysis flowchart used in this study. The general pipeline includes sample preparation and harvesting, sequencing, data analyses, and biological interpretation. The *red highlighted rectangle* illustrates the construction of the *D. tertiolecta* reference library using Bag2D-workflow scripts. Bag2D: Blast1-annotation1-gene model1-Delete redundant genes-Blast2-annotation2-gene model2 (protein name, transcriptID, geneID)

The RNA-Seq data analysis workflow for the non-model species used in this study is illustrated in Fig. 3, which includes upstream cell culturing, harvest and downstream data interpretation steps.

The paired-end reads (130 bps in length/read) from each library were generated using Illumina MiSeq Sequencer. The short paired-end reads from D9, WT were pooled and examined with pre-alignment QA/QC and a secondary pre-alignment QA/QC after trimming was subsequently used. QA/QC reports mentioned throughout the paper are presented in Table 1. The trimmed *D. tertiolecta* short paired-reads were merged and subjected to the assembly programs, Velvet and Oases.

**Table 1:** Run summary on the Illumina MiSeq platform

Sample name	Sequencing stats and pre-alignment QA/QC of raw data/ *pre-alignment QA/QC of post-trimming data*					Post-alignment QA/QC after alignment	
	Total reads	Avg. read length	Avg. read quality	% N	% GC	Total reads	Aligned (%)
D9_S7_L001_R1	954,340/*954,300*	137.28/*137.2*	38/*38.01*	9.62E−04/*0*	47.74/*47.73*	954,300	56.72
D9_S7_L001_R2	954,340/*954,300*	137.07/*136.98*	37.69/*37.7*	1.04E−03/*0*	47.67/*47.66*		
WT_S8_L001_R1	1,084,599/*1,084,546*	136.67/*136.59*	38/*38.01*	1.11E−03/*0*	47.41/*47.4*	1,084,546	57.24
WT_S8_L001_R2	1,084,599/*1,084,546*	136.36/*136.28*	37.76/*37.78*	1.25E−03/*0*	47.34/*47.33*		

*QA/QC* Quality assurance and quality control

Avg. read quality >30 indicating 99.9 % accuracy or greater

The ones in italic font were data generated from post-trimming

### Functional annotation of the genes

Various strategies in optimizing growth conditions and phases have been suggested to stimulate production and accumulation of lipids or starch; these include expression of genes involved in lipids or starch biosynthesis and the maximization of the diversity of expressed genes [7, 9, 22–25]. To verify this and maximize the assembled outputs, sequencing results using different datasets for *de novo* assembly were compared: sequencing data from (1) wild-type only, (2) wild-type and D9 mutant and (3) enlarged Dt database with additional *D. tertiolecta* sample groups (Dt_v1, obtained from a wild-type *D. tertiolecta* strain cultured in a different growth phase, another random insertional mutant from *D. tertiolecta*) were then separately used to generate reference contigs and gene construction. From the comparison in Table 2, the enlarged Dt database Dt_v1 had more hit percentage and transcripts annotation, which was used for the following sections. The command line of BLASTX (from NCBI) was applied to the FASTA data generated from the de novo assembly, to obtain their functional assignments with reference to *C. reinhardtii* protein (Chlre4_best_proteins.fasta, Creinhardtii_169_peptide.fa, Creinhardtii_281_v5.5.protein.fa). By extracting the top hits with a BlastX E value ≤10^−6^, *D. tertiolecta* contigs were annotated. However, there were redundant *D. tertiolecta* contigs assembled with different lengths at distinct positions and junctions. To avoid redundant gene modeling, a filtering step was included with the E-value, confidence of assembled contigs and length of the contigs as the thresholds to select the top hit contig for the unique annotation name. The complete program used here was named as Bag2D-workflow (Blast1-annotation1-gene model1-Delete redundant genes-Blast2-annotation2-gene model2, namely protein name, transcriptID, geneID), using python language based scripts.

**Table 2:** Determining the reference transcripts through de novo assemblies using different sets of data

No.	Samples used for assembly	Size of the assembled file (MB)	Gene model	Number of non-redundant ranscripts
(i)	WT	9.4	3818	14,903
(ii)	WT + D9	16.9	5360	23,564
(iii)	Enlarged Dt-1	45.8	7023	47,276

The BlastX cut-off value is E-value ≤10^−6^ for this comparison. The results of *D. tertiolecta* transcripts with BlastX hits to *C. reinhardtii* as reference

Although the contigs with E-value ≤10^−6^ may not be real transcripts, we decided to keep them to avoid complications with false negatives, accepting that the incidence of false positive could increase. In addition, different cutoff expectation values were set and compared afterwards.

### Comparison of global transcriptome of D9 mutant with wild-type

**Fig. 4:**
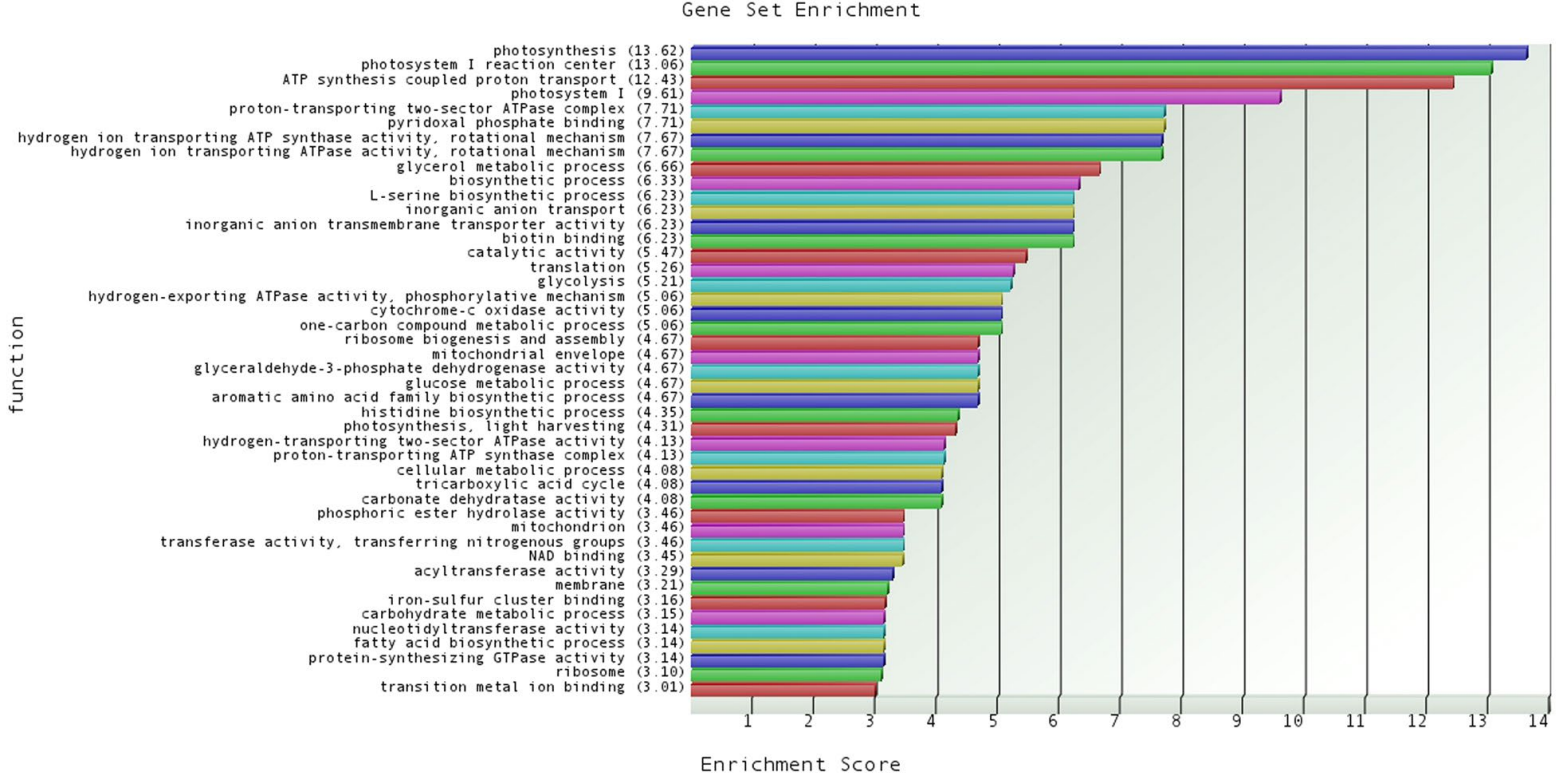
GO enrichment result of up-regulated genes in D9 mutant compared to wild-type *D. tertiolecta.* If a functional group has an enrichment score over 1, functional category is over expressed. A value of 3 corresponds to significant over expression (*p*-value of less than 0.05)

The gene specific analysis generated from Partek Flow was imported into Partek Genomics Suite for statistical analyses. The data quality of log2-transformed RPKM values for these two RNA-Seq datasets was checked using the sample histogram. The peak and trend of the curves indicated that the normalization method was suitable for this dataset and the RNA-Seq data were of high quality and similarly distributed. Gene list was created with threshold of false discovery rate (FDR) ≤0.05, multiple fold change (FC) ≥1.5 or FC ≤ −1.5 (to detect as many genes as possible that is different between the mutant and WT) and subsequently merged with the above generated annotation file with the common column of *D. tertiolecta* contig names (results were reported in Additional file 2). The annotated gene list created was used for further biological interpretation. Gene ontology (GO) enrichment categories also provided insights into differentially distributed protein functional families and categories. The up-regulated GOs were highly represented in the photosynthesis and ATP synthesis families, as shown in Fig. 4. Fatty acid synthesis requires stoichiometric amount of ATP, acetyl CoA and NADPH for each two carbon added to the growing acyl chain [26]. Light-driven electron transport is coupled to ATP synthesis in chloroplasts [27]. Photosynthetic reactions are thus essential not only in providing a carbon source but also in generating reducing power (NADH and NADPH) and energy (ATP) for fatty acid synthesis [26]. According to this, mechanisms for enhanced lipid production could be elucidated, while comparable growth rate was observed between D9 mutant and WT cultures.

The KEGG pathway analysis showed that inositol phosphate metabolism, fatty acid biosynthesis, biosynthesis of secondary metabolites, fatty acid metabolism and purine metabolism were significantly correlated with higher lipid yield based on the cutoff of enrichment *p* value less than or equal to 0.05 (Additional file 3). From the pathway analysis, the top two pathways with low-abundance contigs were the inositol phosphate metabolism and fatty acid biosynthesis pathways.

From the inositol phosphate metabolism, the gene that encodes myo-inositol oxygenase (MIOX) [EC: 1.13.99.1] showed significant up-regulation, which led to enhanced generation of D-Glucuronate as the substrate for both ascorbate acid synthesis pathway and generation of D-Glucarate. Fatty acid biosynthesis was the second top hit pathway. The up-regulated gene coding for ACCase [EC: 6.4.1.2] was detected in spite of a down-regulation of the gene (*FabG*) coding for 3-oxoacyl-[acyl-carrier protein] reductase [EC: 1.1.1.100], which is involved in the fatty acid chain elongation reactions.

**Fig. 5:**
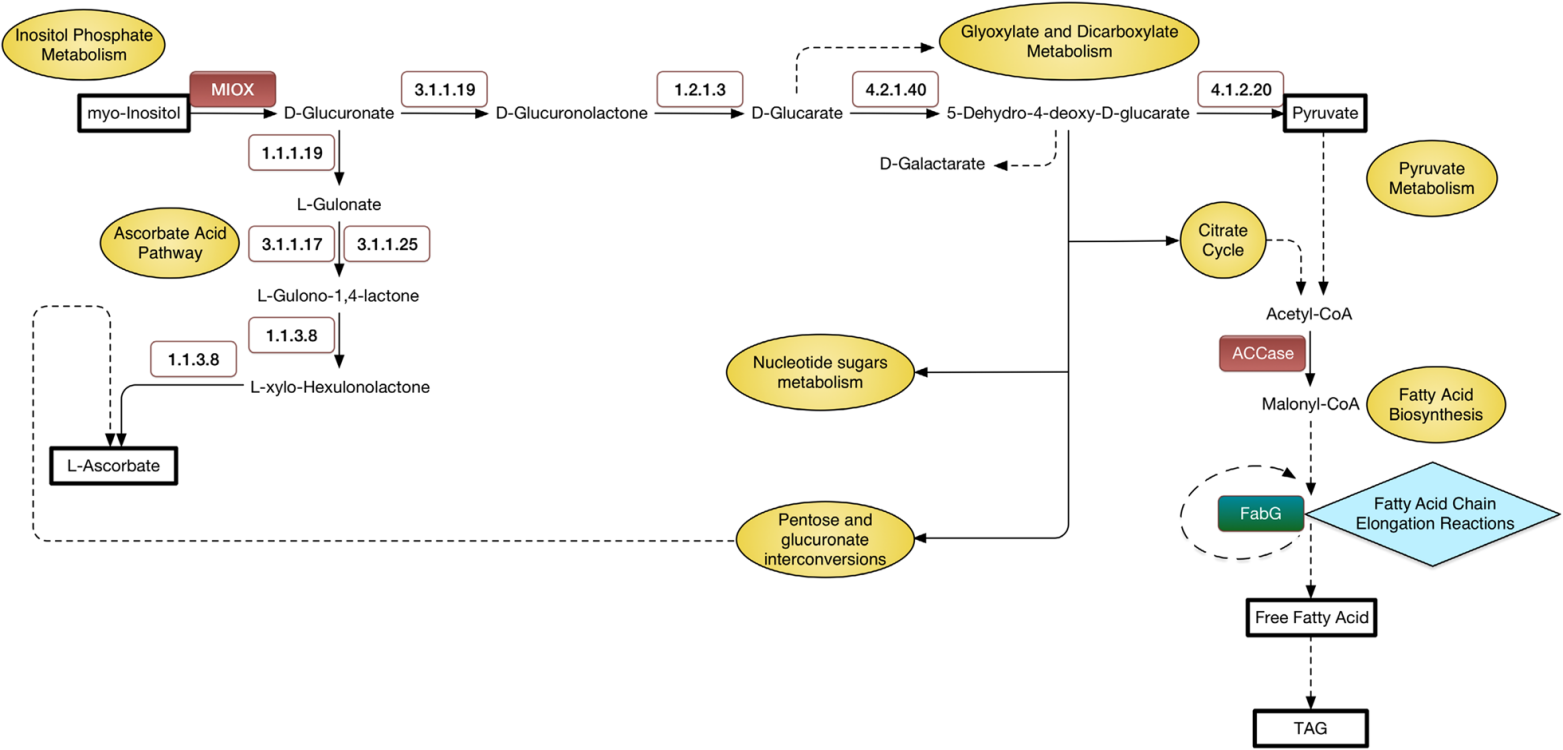
Pathway analyses for mutant D9 and wild-type *D. tertiolecta.* Inositol phosphate metabolism, ascorbate acid synthesis, citrate cycle, pyruvate metabolism, and fatty acid biosynthesis pathways in *D. tertiolecta* constructed based on the information from *C. reinhardtii* and the transcript abundance of enzymes in *D. tertiolecta*. Key components are represented in *black rectangle boxes*. *The green color* indicates that the respective gene is down-regulated, and *red color* indicates up-regulation. The corresponding pathways are also represented in the *yellow filled circles*

In Fig. 5, we attempted to correlate the transcriptomic data with metabolite pathways. Changes within the metabolic intermediates with the metabolic pathways between D9 mutant and wild-type *D. tertiolecta*, namely the inositol phosphate metabolism, ascorbate acid synthesis, citrate cycle, pyruvate metabolism and fatty acid biosynthesis pathways were reconstructed based on the information from *C. reinhardtii* ChlamyCyc database [28] and the transcript abundance of enzymes in *D. tertiolecta*.

It is noted that the horizontal branch in the pathways in Fig. 5 goes into the production of pyruvate. No significant differentially expressed genes in glyoxylate and dicarboxylate metabolism were identified in D9 mutant as compared to WT.

Up-regulation of the gene coding for ACCase was observed. As ACCase catalyzes the first reaction of the fatty acid biosynthesis pathway, namely the formation of malonyl CoA from acetyl CoA and CO_2_, its up-regulation may channel carbon flux to fatty acid synthesis. This suggests that concurrent over-expression of genes particularly in ACCase as the first committed step along the Inositol Phosphate Pathway and the Fatty Acid Biosynthesis Pathway will enhance neutral lipid production in algae.

To form a saturated fatty acid, the 3-ketoacyl ACP product is reduced by the enzyme FabG [29]. Herein, we predict that the down-regulation of the gene coding for FabG in the D9 mutant may change the length and saturation level of the fatty acid chain, leading to accumulation of more short-chain fatty acids or unsaturated fatty acids. The 16- or 18-carbon fatty acids are formed by a series of two-carbon chain elongating reactions catalyzed by a multi-subunit enzyme in most plants and algae [30]. Viscosity increases with fatty acid chain length [31] so that most plant TAGs have a viscosity range that is much higher than that of conventional diesel [32]. The higher viscosity results in poor fuel atomization in modern diesel engines, leading to problems derived from incomplete combustion such as carbon deposition and coking [33]. Medium-chain fatty acids (C8–C14) are favorable for production of biofuels, for they have properties that mimic current diesel fuels and they improve fluid characteristic of the liquid fuel [30, 34].

Pre-analysis for an additional D9 biological duplicate RNA-Seq data for validation was presented in Additional file 4. With more stringent criteria of FC ≥ 2 or FC ≤ −2 and FDR ≤ 0.05, we found an overall similar pattern from gene expression profiles and KEGG pathway analysis results in Additional files 5 and 6. The expression levels of many genes in the metabolic pathways, photosynthesis pathways, carbon fixation pathways and fatty acid pathways were increased. Two randomly selected genes showed similar expression patterns in both real-time PCR and RNA-Seq (Additional file 7). This batch of experiment gave us a good hint that our results from the previous RNA-Seq experiment are reproducible.

### Comparison of *D. tertiolecta* transcriptome database with other microalgae

Contigs from aforementioned Dt-1 database were compared to the transcripts/proteins from five other chlorophyta species with known genome sequences (*C. reinhardtii, C. subellipsoidea* C-169*, O. lucimarinus, V. carteri* and *C. variabilis* NC64A) by BlastN/BlastX. Compared with BlastX results, BlastN results retrieved fewer hits (Table 3). For example, 7023 *D. tertiolecta* contigs matched *C. reinhardtii* proteins using BlastX with a cut-off E-value of 10^−6^ indicating 35.97 % of the *C. reinhardtii* proteins could be assigned in *D. tertiolecta,* while only 611 (3.13 %) *D. tertiolecta* contigs matched *C. reinhardtii* using BlastN with cut-off E-value of 10^−6^. Similar patterns were also observed in the Blast results from the other three green algae at cut-off E-values of 0, 10^−10^, 10^−6^, 10^−3^, respectively. This result indicates that the sequences of microalgae are more conserved at the protein level than that at the nucleotide level; therefore, BlastX results were subsequently used in this study for gene annotation.

**Table 3:** Comparisons of homologues between *D. tertiolecta* and other five green algae with reference genome sequences

Algal reference	*Cre*	*Csu*	*Olu*	*Vca*	*ChlN*
Reference Transcripts or proteins #	19,526	9629	7796	15,285	9791
BlastN					
Transcript hit (E-value = 0)	82 (0.42 %)	18 (0.19 %)	0 (0.00 %)	24 (0.16 %)	20 (0.20 %)
Transcript hit (E-value ≤10^−10^)	574 (2.94 %)	229 (2.38 %)	15 (0.19 %)	246 (1.61 %)	281 (2.87 %)
Transcript hit (E-value ≤10^−6^)	611 (3.13 %)	249 (2.59 %)	16 (0.21 %)	269 (1.76 %)	307 (3.14 %)
Transcript hit (E-value ≤10^−3^)	654 (3.35 %)	252 (2.62 %)	16 (0.21 %)	278 (1.82 %)	315 (3.22 %)
BlastX					
Transcript hit (E-value = 0)	583 (2.99 %)	356 (3.70 %)	205 (2.63 %)	523 (3.42 %)	239 (2.44 %)
Transcript hit (E-value ≤10^−10^)	6610 (33.85 %)	5025 (52.19 %)	4002 (51.33 %)	6249 (40.88 %)	5106 (52.15 %)
Transcript hit (E-value ≤10^−6^)	7023 (35.97 %)	5643 (58.60 %)	4592 (58.90 %)	6986 (45.70 %)	5773 (58.96 %)
Transcript hit (E-value ≤10^−3^)	8561 (43.84 %)	6393 (66.39 %)	5267 (67.56 %)	8162 (53.40 %)	6604 (67.45 %)

The numbers before and within brackets are the number of *D. tertiolecta* transcripts with Blast hits from reference alga and the hitting percentage in the corresponding reference transcripts/proteins

*Cre*, *C. reinhardtii*; *Csu*, *C. subellipsoidea*; *Olu*, *O. lucimarinus*; *Vca*, *V. carteri*; *ChlN*, *C. variabillis* N64A

**Fig. 6:**
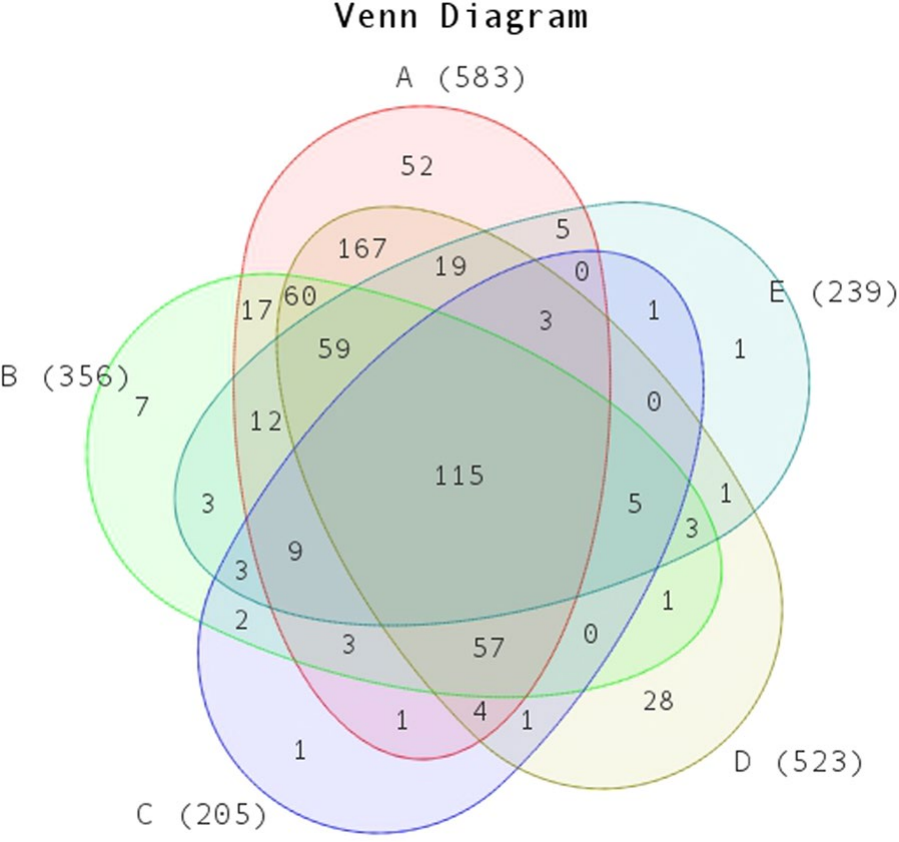
Venn diagram of the numbers of *D. tertiolecta* transcripts with BlastX hits from five model algae. The BlastX cut-off value is 0 for this comparison.* A* The number of *D. tertiolecta* transcripts with BlastX hits to *C. reinhardtii*;* B* The number of *D. tertiolecta* transcripts with BlastX hits to *C. subellipsoidea* C-169;* C* The number of *D. tertiolecta* transcripts with BlastX hits to *O. lucimarinus*;* D* The number of *D. tertiolecta* transcripts with BlastX hits to *V. carteri*; and* E* The number of *D. tertiolecta* transcripts with BlastX hits to *C. variabilis* NC64A

**Fig. 7:**
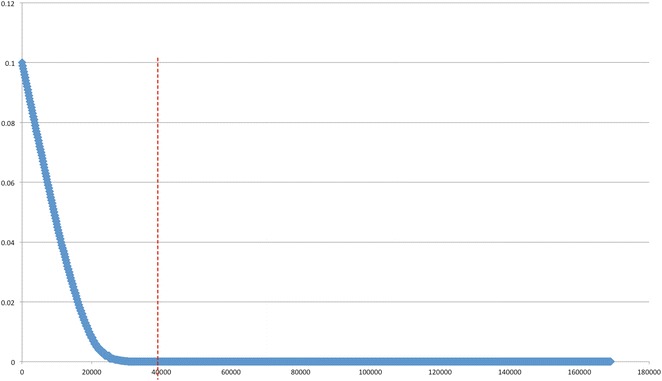
Length distributions of the contigs from Dt_v10. X-axis: number of the contigs. Y-axis: E-value of the contigs

With the decrease of E-value stringency from 0 to 10^−10^, 10^−6^ and 10^−3^, the BlastX hit number for each transcript increased and the percentage of total contigs matching reference sequences increased (Table 3). To exclude possible false positives, only those contigs with E-value of 0 from BlastX results were used for identification of *D. tertiolecta* true genes. The results indicate that these six green algae have 115 contigs in common, and a total of 640 genes in *D. tertiolecta* could be annotated and identified (the overlaps and unique areas in the Venn diagram in Fig. 6) in the five references, while 583 contigs were in common with *C.reinhardtii*. From the Venn diagram in Fig. 6, some *Dunaliella* genes could not match that of the *Chlamydomonas* genes, but they do overlap with genes from the other four model algae. Through a comparison study on E-value from 0 to 0.1, the number of corresponding transcripts was plotted out in Fig. 7, and 10^−6^ was used as the threshold from the distribution of the hitting contigs. Therefore, a modification process was applied with a combination of identified *D. tertiolecta* transcripts from the BlastX results with the above five green algae, by increasing E-value number to 10^−6^ BlastX for the construction of *D. tertiolecta* database.

### Validation and optimization of the *D. tertiolecta* transcriptomic database

To validate the accuracy of the *D. tertiolecta* transcriptomic database from Bag2D, reported *D. tertiolecta* nucleotides on National Center for Biotechnology Information (NCBI) were compared. The transcripts with the same *Chlamydomonas* protein annotation were compared in Table 4. The one with a higher lower E-value compared to the reference species will be used in the final *D. tertiolecta* database. From such comparison, we found that most contig sequences from Bag2D were identical compared to the NCBI published ones, as concluded from the high alignment scores generated from multiple alignment tools. Though we found some low alignment score pairs, which might caused by the existence of isoforms or errors of the online available sequences. Besides, we found there are some redundant nucleotides, like gi|46981381|gb|AY575952.1| *Dunaliella tertiolecta* assimilatory nitrate reductase (nar) gene (partial cds), gi|311818483|emb|HH768845.1| Sequence 5032 from Patent EP2221382, gi|18913154|gb|AY078279.1| *Dunaliella tertiolecta* assimilatory nitrate reductase (nar) mRNA (complete cds), with different NCBI ID numbers and names, which might cause misunderstanding. Some new genes have been found and the sequences are available, e.g., acetyl-coa carboxylase beta-carboxyltransferase subunit of plastidic multimeric ACCase, with the name of >Locus_2576_1Transcript_1/1_Confidence_1.000_Length_1955. The *D. tertiolecta* database was optimized with a better sequence or the original sequence elongated.

**Table 4:** Comparison studies of transcripts from Bag2D program and NCBI

*D. tertiolecta* transcripts from Bag2D	E-value	Length (bps)	*D. tertiolecta* nucleotide from NCBI	E-value	Length (bps)	Aligned score	Elongated
>Locus_1123_4Transcript_1/2_Confidence_0.800_Length_1500	0	1500	>gi|46981381|gb|AY575952.1| Dunaliella tertiolecta assimilatory nitrate reductase (nar) gene, partial cds	2e−18	1313	–	
			>gi|311818483|emb|HH768845.1| Sequence 5032 from Patent EP2221382	0	2531	90.6	
			*>gi|18913154|gb|AY078279.1| Dunaliella tertiolecta assimilatory nitrate reductase (nar) mRNA, complete cds*	0	3447	90.6	+
*>Locus_96_4Transcript_1/1_Confidence_1.000_Length_1881*	0	1881	>gi|3869303|gb|AF065142.1| Dunaliella tertiolecta glutamine synthetase mRNA, partial cds	2e−109	601	92.8453	
*>Locus_797_6Transcript_1/1_Confidence_1.000_Length_1463*	1e−144	1463	>gi|2645974|gb|AF034201.1| Dunaliella tertiolecta proliferating cell nuclear antigen (PCNA) mRNA, partial cds	7e−122	616	97.8896	
*>Locus_6018_7Transcript_2/2_Confidence_0.667_Length_959*	2e−115	959	>gi|12232559|gb|AF036312.2| Dunaliella tertiolecta mitotic cyclin mRNA, partial cds	6e−47	490	96.1224	+
*>Locus_1375_7Transcript_7/9_Confidence_0.632_Length_1220*	1e−132	1220	>gi|167984|gb|M60049.1|DUNCAB D.tertiolecta 28.5-kDa LHCII apoprotein mRNA, complete cds	2e−132	1041	91.0663	
*>Locus_169_2Transcript_3/5_Confidence_0.632_Length_1062*	3e−122	1062	>gi|225322931|gb|FJ769282.1| Dunaliella tertiolecta ascorbate peroxidase mRNA, partial cds	1e−72	546	87.5458	
*>Locus_1725_6Transcript_1/1_Confidence_1.000_Length_1811*	0	1811	>gi|585087513|gb|KF193066.1| Dunaliella tertiolecta sedoheptulose-1,7-bisphosphatase (SBP) mRNA, complete cds	2e−180	1631	97.9767	
>Locus_613_6Transcript_1/1_Confidence_1.000_Length_1405	0	1405	*>gi|682124627|gb|KJ930518.1| Dunaliella tertiolecta mitogen-activated protein kinase (MAPK) mRNA, complete cds*	0	1687	90.3915	+
>Locus_284_4Transcript_1/1_Confidence_1.000_Length_1355	2e−16	1355	*>gi|371532816|gb|JQ039042.1| Dunaliella tertiolecta ATP-dependent Clp protease proteolytic subunit (clpP) mRNA, complete cds; plastid*	2e−16	1560	98.2288	
>Locus_17606_9Transcript_1/1_Confidence_1.000_Length_2042	0	2042	*>gi|682124625|gb|KJ930517.1| Dunaliella tertiolecta phosphofructokinase (PFK) mRNA, complete cds*	0	2049	99.3144	
*>Locus_979_2Transcript_1/2_Confidence_0.833_Length_2662*	4e−139	2662	>gi|144601644|gb|EF471039.1| Dunaliella tertiolecta isolate 1 NRT2 (Nrt2) gene, partial cds	5e−50	644	94.4099	
>Locus_1062_4Transcript_1/1_Confidence_1.000_Length_630	2e−82	630	*>gi|19879329|gb|AY032598.1| Dunaliella tertiolecta nucleoside diphosphate kinase mRNA, complete cds*	7e−57	840	42.5397	*
*>Locus_2576_1Transcript_1/1_Confidence_1.000_Length_1955*	0	1955	>gi|459938225|gb|KC572136.1| UNVERIFIED: Dunaliella tertiolecta isolate PL1 acetyl-CoA carboxylase beta subunit-like (accD) gene, partial sequence; chloroplast	1e−42	925	44.973	
*>Locus_468_7Transcript_1/1_Confidence_1.000_Length_1448*	0	1448	>gi|4165328|gb|AF038570.1| Dunaliella tertiolecta cyclin-dependent kinase 1 (DUNCDC2) mRNA, complete cds	0	1061	99.7172	
>Locus_652_4Transcript_1/1_Confidence_1.000_Length_2435	0	2435	*>gi|371532796|gb|JQ039032.1| Dunaliella tertiolecta ATP synthase CF1 alpha subunit (atpA) mRNA, complete cds; plastid*	0	1515	42.8383	*
*>Locus_7608_9Transcript_1/1_Confidence_1.000_Length_2170*	0	2170	>gi|371532798|gb|JQ039033.1| Dunaliella tertiolecta ATP synthase CF1 beta subunit (atpB) mRNA, partial cds; plastid	5e−177	1167	46.8723	*
*>Locus_3958_1Transcript_1/1_Confidence_1.000_Length_2042*	0	2042	>gi|371532912|gb|JQ039091.1| Dunaliella tertiolecta elongation factor Tu (tufA) mRNA, complete cds; plastid	8e−154	1257	47.494	
*>Locus_3579_4Transcript_1/1_Confidence_1.000_Length_943*	1e−64	943	>gi|371532800|gb|JQ039034.1| Dunaliella tertiolecta ATP synthase CF1 epsilon subunit (atpE) mRNA, complete cds; plastid	3e−07	408	26.4706	*
*>Locus_6870_1Transcript_3/3_Confidence_0.778_Length_1870*	1e−117	1870	>gi|761262915|gb|KJ930371.1| Dunaliella tertiolectaglucose-6-phosphate dehydrogenase (G6PDH) mRNA, complete cds;chloroplast	3e−72	3246	34.6524	*

BlastX with *C. reinhardtii* using cut-off value at E-value ≤10^−6^ for this comparison

The one in italic font was chosen in the database optimization for each gene

+, the NCBI nucleotide was chosen for the corresponding gene, but it was elongated by part of the sequence from Bag2D

*, the two nucleotides did not quite match from the two methods, which may have been caused by the existence of isoforms or errors of the one from NCBI (determined by E-value and analyses of the sequences)

**Fig. 8:**
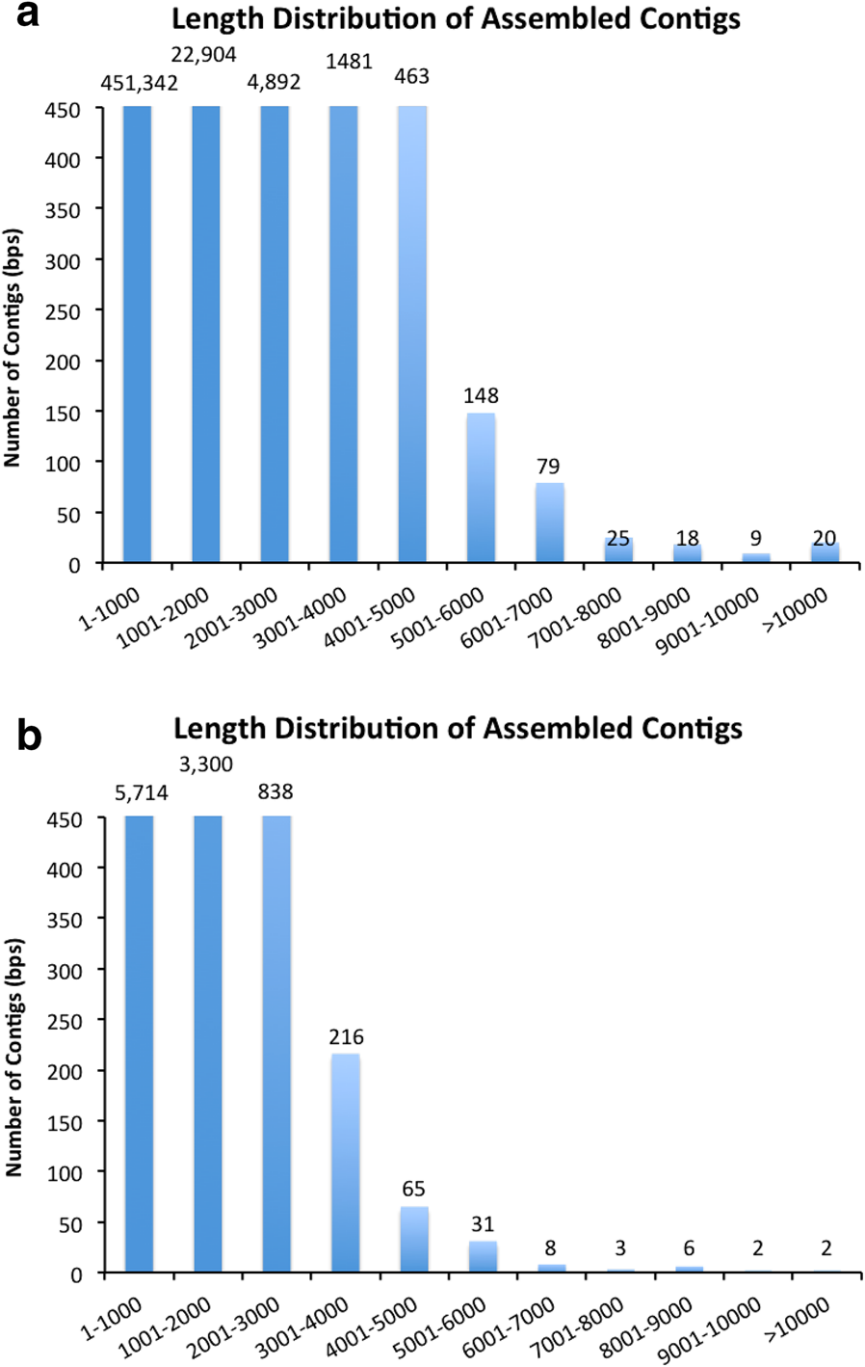
Length distribution of the contigs in Dt_v10 and Dt_v10-hit. **a** Statistical results of the length of *D. tertiolecta* reference contigs after assembly and filtering, total 181Mega-base nucleotides (481,381 contigs) with minimum length of 74 bps, maximum length of 17,995 bps, and average length of 376 bps. **b** Statistical results of the length of *D. tertiolecta* reference contigs after assembly and filtering, total 11 Mega-base nucleotides (10,185 contigs) with minimum length of 101 bps, maximum length of 15,975 bps, and average length of 1106 bps

We optimized the *D. tertiolecta* transcriptome database mainly by two strategies: (1) Enlarging the *D. tertiolecta* transcriptome sequence in the database. Miseq output data after each run were included in the latest version and spiked with all the reported *D. tertiolecta* nucleotide sequences from NCBI to generate Dt_v10 after running through Bag2D pipeline and (2) annotating the *D. tertiolecta* sequence with an addition of model high plant species. Considering the functional annotation of some microalgae may be problematic because annotation is largely based on plants and there is large phylogenetic distance between them. We also compared *D tertiolecta* with a high-quality annotation of plant species *Arabidopsis thaliana*, using the latest large Dt merged database (Dt_v10). As a result, Dt_v10 database has a total 181 Mega-base nucleotides (481,381 contigs) ranging from 74 bps to 17,995 bps in length that were obtained for RNA-Seq alignment. The length distribution of the assembled transcripts was described in Fig. 8. Dt_v10-hit database (only including the contigs that can be annotated in Dt_v10 by 6 reference species) has a total 11 Mega-base nucleotides (10,185 contigs) with minimum length of 101 bps, maximum length of 15,975 bps and average length of 1106 bps (7052 was generated from *C. reinhardtii* and for RNA-Seq analysis). This allows us to identify a good quantity of *D.tertiolecta* gene sequences.

### *Chlamydomonas* RNA-Seq data were used as the benchmark

We used a set of *Chlamydomonas reinhardtii* RNA-Seq data as the benchmark to check the performance of Bag2D program and Partek software. Since *Chlamydomonas* has the genome sequence annotated, it can be performed in the standard way in the Partek Flow software for the alignment and annotation. It can also be assembled and annotated using in-house Bag2D software. From Additional file 8, we found 88 % of the pathways were hit in both methods. A comparison of the top hit KEGG pathway generated from both methods was presented in Additional file 9, where a similar hit pattern of the over-representative genes in both methods were found.

## Conclusions

This study provided a workflow (Bag2D) to construct a transcriptome database for the analysis of RNA-Seq data on the lipid-rich mutant. We have generated a lipid-rich microalgal mutant, named D9, via random mutagenesis. To characterize it on the transcriptomic level, RNA-Seq technology was applied and Bag2D was developed. After being validated in a model algal species *Chlamydomonas* as the benchmark, Bag2D is currently free for public access. It could serve as a model tool to construct transcriptome databases for those organisms without complete transcriptome information. Similarly, Hamid et al. [9] presented the first next-generation sequencing effort and transcriptome annotation of a non-model marine microalgae that is relevant to biofuel production by extracting cells from various growth conditions and different phases of growth cycle, using the 454 Genome Sequencer FLX with Titanium Chemistry. As a result, they identified 33,307 assembled isotigs, 409,789 unique isotigs and singletons and 8466 isotigs within E-value threshold of 10^−6^. Despite their achievement, using Bag2D-workflow to analyze the RNA-Seq data from Illumina Miseq, we could obtain and identify more assembled contigs (181.2 MB) and unique transcripts (10,185 transcripts, with E-value threshold of 10^−6^) with functional assigned.

From the RNA-Seq result, it can been seen that elimination of the gene (*FabG*) coding for 3-oxoacyl-[acyl-carrier protein] reductase can relieve the feedback inhibition of β-ketoacyl-ACP synthase (*fabB* or *fabH*) caused by the accumulation of fatty acyl-ACPs [35] and terminate the chain elongation cycle [19], along with the boosting of rate-limiting precursor (malonyl-CoA) flux by the expression of the acetyl-CoA carboxylase (ACCase), which leads to the D9 mutant producing about 2- to 4-fold more fatty acids. The upstream enzyme myo-inositol oxygenase was overexpressed to convert more precursors for the biosynthesis of fatty acid. In addition, from KEGG pathway analysis and GO enrichment analysis, many photosynthesis-related genes were found to be overexpressed. Photosynthetic rate measurement also proved that more energy was generated from photosynthesis in the mutant. Based on the results, it appears that this workflow is able to provide biological insights into the expression patterns of transcripts associated with energy metabolism and carbon flow pathways. This multiple pathway engineering approach that focuses on optimizing the expression of several related pathways is potentially useful for gene manipulation that can be directly applied to engineering microalgal fatty acid production.

## Methods

### Culture strains and culture condition

*Dunaliella tertiolecta* strain UTEX LB-999 was obtained from the UTEX Culture Collection of Algae (University of Texas at Austin, TX, USA). *D. tertiolecta* cells were grown in flasks in sterile ATCC-1174 DA liquid medium (American Type Culture Collection at Manassas, Virginia) containing 0.5 M NaCl incubated on a rotary shaker at 25 °C, under 12 h light/12 h dark with light intensity of 30 μmol photons m^−2^ s^−1^. For the purpose of clarity, work conducted throughout the paper is based on biological and technical triplicates unless otherwise stated.

### *Dunaliella tertiolecta* transformation and mutant screening

The pGreenII0000 plasmid incorporated a 431-bps *CrRBCS2* promoter and a 526-bps bleomycin resistance gene (*ble*) to confer zeocin-resistance [36] was designed and constructed in our laboratory.

*D. tertiolecta* cells were transformed using the glass bead method [37] with minor modifications. Cultures grown for 5 days in sterile ATCC-1174 DA liquid medium containing 0.08 M NaCl were harvested by centrifugation (3000×*g*, 10 min) and resuspended in 0.08 M NaCl ATCC-1174 DA liquid medium to a cell concentration of 1–2 × 10^8^ cell mL^−1^. A 300-μl aliquot of this cell suspension was added to a 12-mL round-bottom tube containing 0.3 g acid-washed glass beads (425–600 μm diameter; Sigma), 100 μl 20 % polyethylene glycol (PEG-8000; Sigma), 1 μg linearized plasmid and 5 μl fish sperm DNA. After vortexing for 20 s at maximum speed, the cells were plated immediately onto 0.08 M NaCl ATCC-1174 DA medium agar plates containing 10 μg mL^−1^ zeocin (Invitrogen). Colonies that appeared within 3 weeks were picked and inoculated into 0.5 M NaCl ATCC liquid medium. Secondary selection in 0.08 M NaCl ATCC medium with 5 μg mL^−1^ zeocin was performed to reduce the false-positive transformants.

### Determination of cell growth and quantitative lipid measurement

Cell concentration was counted twice a day to monitor the growth of the culture. Stationary phase was reached when the cell concentration and optical density (OD_680_) remained constant. The growth kinetic study was repeated at least three times.

Fatty acid methyl esters (FAMEs) were prepared by direct transmethylation with sulfuric acid in methanol [38]. The FAMEs were analyzed by gas chromatograph (model 7890B, Agilent Technologies) equipped with a mass-selective detector (model 5977A, Agilent Technologies) [39, 40].

Nile red staining quantification method offers an indirect measurement for lipids, binding specifically to neutral lipids, which is the most commonly used lipophilic stain for intracellular TAG detection in microalgae [41]. Nile Red measurement was found to parallel that of the GC measurement, and since Nile Red method is a rapid, we mainly measured lipid by Nile Red and report here using Chen’s protocol [42] with minor modification. Cells were collected every 2 days from mutants and wild-type *D. tertiolecta*. Cell concentration was estimated using OD_680_. Cells were harvested by centrifugation at 3000×*g* for 10 min and resuspended in fresh ATCC-1174 DA liquid medium with the same salinity, to an OD_680_ of 0.3. Two hundred microliters of cell samples, culture medium blank and triolein standard were transferred to a 96-well black, clear-bottom plate. The plate was read at the excitation and emission wavelength at 524 nm and 586 nm, respectively, before and after addition of 2 μl Nile red working solution prepared in acetone [42] and incubated for 5 min in the dark.

### Determination of photosynthetic rate and photosynthetic efficiency

The mutants and wild-type *D. tertiolecta* cells were collected on day 15 by centrifugation at 12,000×*g* for 15 min at 4 °C. The high centrifugal force served to weaken the cell structures to facilitate extraction of chlorophyll. Chlorophyll content of individual sample was estimated spectrophotometrically [43]. Subsequently, photosynthetic performance was carried out using an oxygen electrode according to the operating manual (Rank Brothers, Bottisham, Cambridge, UK).

### Determination of glycerol and starch content

One milliliter of the D9 and wild-type *D. tertiolecta* liquid culture at stationary phase (day 15) was harvested for the determination of intra- and extracellular glycerol contents. The level of glycerol was determined using Free Glycerol Determination Kit (Sigma FG0100), according to the protocol provided by the manufacturer. Ten milliliters of the *D. tertiolecta* cells at stationary phase was collected and the starch content was determined using Starch Assay kit (Sigma STA20), according to the instructions provided by the manufacturer.

### Construction of cDNA libraries for next-generation sequencing analysis

Total RNA of D9, WT was extracted on day 8 from *D. tertiolecta* cells using an RNeasy^®^ Plant Mini Kit (Qiagen, Valencia, CA, USA), according to manufacturer’s protocol. Approximately 2 μg of the resulting total RNA was used for synthesis of cDNA using the TruSeq Stranded Total RNA LT Sample Prep Kit (with Ribo-Zero Plant) (Illumina) according to manufacturer’s instruction including synthesis of first and second strands cDNA, end repair, 3′-end adenylaton, adapter ligation, fragment enrichment (e.g., ~350 bps, in length), library validation and quantification. The libraries were sequenced using Illumina MiSeq Sequencer (Illumina, Inc., San Diego, CA, USA) according to the manufacturer’s instructions. To check the variance from the effect of RNA-Seq technology on the mutant and wild-type samples, we extracted another set of duplicate samples on Day 12, and the same process was carried out as the previous experiment group.

### De novo assembly of Illumina short reads and RNA-Seq data processing

The FASTQ datasets of *D. tertiolecta* generated from Illumina MiSeq were imported into Partek^®^ Flow^®^ software (version 3.0 Copyright©; 2014 Partek Inc., St. Louis, MO, USA) for quality assessment. The raw data were then trimmed from both ends based on the following parameter setting: min read length = 25; quality encoding = auto detect; end min quality level (Phred) = 20. *D. tertiolecta* database was *de novo* assembled via Velvet and subsequently Oases assembler. The parameters used for Velvet assembly were as follows: hash length = 21; expected coverage = 10; max coverage = 500; min coverage = 1; min contig length = 50; min long cutoff = 2; max branch length = 100; max divergence rate = 0.2; max gap count = 3; min read-pair validation = 10. The parameters used for Oases assembly were as follows: coverage cutoff = 3; min paired cutoff = 4; min observed to estimate ratio = 0.01; edge sensitivity = 0.01; contig uniqueness = 3. A user-friendly script count_geneLength.py was used to determine the length of the assembled contigs.

The paired-end reads were mapped back to the assembled contigs. Data aligned to the transcriptome in RNA-Seq analysis were selected to estimate transcript abundance. Gene-specific analysis was subsequently used to compare the samples from mutant D9 and WT at transcript level using the default setting (Poisson model was used). Read hits per contig were normalized to RPKM (Reads Per Kilobase per Million mapped reads) used for estimation at transcription level. One-way ANOVA analysis was used for the differentially expressed transcripts in at least one comparison for day 12 RNA-Seq data.

### Comparisons among other green algae and high plant species

The FASTA files of transcripts and proteins sequence of *C. reinhardtii*, *V. carteri*, *C. subellipsoidea* C-169, *C. variabilis* NC64A were downloaded from JGI website (http://www.jgi.doe.gov) and *Arabidopsis thaliana* from the Arabidopsis Information Resource (http://www.arabidopsis.org) and used as reference sequences for alignment with *D. tertiolecta* transcripts as query sequences. The *D. tertiolecta* hit profiles among different species were compared.

### Optimization of the *D. tertiolecta* database and Bag2D program

To make the *D. tertiolecta* database more complete and accurate, more Miseq RNA-Seq data were included together with the NCBI published sequence information. Since the Bag2D program is in-house constructed, we validate it using a set of RNA-Seq data from *Chlamydomonas* as a benchmark.

### Functional annotation of the *D. tertiolecta* contigs and biological interpretation

*D. tertioleta* is a species that has not been sequenced or annotated. Using the reference genome of related species such as *C. reinhardtii* or *V. carteri* or *C. subellipsoidea* C-169, and *C. variabilis* NC64A, the percentages of hit for *D. tertiolecta* in Partek Flow STAR aligner were less than 5 % (data not shown). To improve the hits, a novel Bag2D-workflow program using python language based scripts was developed. A typical run of Bag2D consists of two steps. From the precursor *de novo* assembly that was obtained from the Partek Flow using Velvet + Oases, a set of blast output was generated by BlastX using the query information of *D. tertiolecta* transcript list and the reference protein sequences of related species with a customer e value of E-value ≤10^−6^. Subsequently, *D. tertiolecta* contigs were selected from the top hits, and genes with reference to the model organisms were constructed. The redundant transcripts were filtered using a “delete” program. With the remaining *D. tertiolecta* contigs, BlastX was conducted for a second time. The corresponding gene ID, transcript ID and protein name were sorted and the resulting files were used as a new ‘annotation’ file (on the author’s Github page) to perform further gene analysis and biological interpretation by Partek^®^ Genomics Suite^®^ software (version 6.6 Copyright©; 2014 Partek Inc., St. Louis, MO, USA). The two steps were carried out by the user-friendly scripts extract_blast.py, and get_geneID/transcriptID/proteinname.py (including count_geneLength.py). The steps of the Bag2D-workflow appear as red box in Fig. 3. The gene-specific analysis was subsequently imported into PGS with the annotation file. They were merged according to the name of *D. tertiolecta* contigs. The annotated data were used to perform GO enrichment with a modified *C. reinhardtii* GO annotation file downloaded from JGI website (http://www.jgi.doe.gov), using Fisher’s Exact test with a restrict analysis of more than two genes. The representative pathways were generated using pathway analysis tool, by Fisher’s Exact test and the *C. reinhardtii* KEGG database (http://www.genome.jp/). The novel Bag2D program package and computationally processed *Dunaliella tertiolecta* draft transcriptome database and its annotation files are hosted at the author’s GitHub page https://github.com/SPURc-Lab/NGS-D9 with the step-by-step user manual for public access. The raw data from RNA-Seq and processed files were deposited into GEO with accession number of GSE70876. We have made the simulation data for this experiment available on the website.

## Additional files

10.1186/s13068-015-0382-0 Linear regression of Nile red assay and GC–MS measurement. Horizontal error bars show standard deviation for Nile red assay; Vertical error bars show standard deviation for GC–MS assay. The line shows linear regression between the two methods. Strain genotypes 1, 3, and 5 represent culture days 8, 13, and 16 for wild-type strain, respectively; 2, 4, and 6, represent culture days 8, 13, and 16 for D9 mutant strain. All experiments were performed in triplicate.
10.1186/s13068-015-0382-0 D9 and wild-type *Dunaliella tertiolecta* RNA-Seq result.
10.1186/s13068-015-0382-0 D9 and wild-type *Dunaliella tertiolecta* KEGG pathway analysis results of RNA-Seq first run. The top hit pathways in pathway analysis with p value ≤0.05.
10.1186/s13068-015-0382-0 Run summary of second round of D9 duplicate samples on the Illumina MiSeq platform.
10.1186/s13068-015-0382-0 D9 and wild-type *Dunaliella tertiolecta* One-way ANOVA results of RNA-Seq second run.
10.1186/s13068-015-0382-0 D9 and wild-type *Dunaliella tertiolecta* KEGG pathway analysis results of RNA-Seq second run. The top hit pathways in pathway analysis with p value ≤0.05.
10.1186/s13068-015-0382-0 Comparison of gene expression profiles from real-time PCR and RNA-Seq. a) The ratio of Locus_1462_2Transcript_1/2 expression level in D9 compared to wild-type *D. tertiolecta* is 7.8 from qPCR and 9.6 from RNA-Seq. b) The ratio of Locus_15808_1Transcript_1/1 expression level in D9 compared to wild-type *D. tertiolecta* is 6.2E − 04 from qPCR and 1.1E − 06 from RNA-Seq.
10.1186/s13068-015-0382-0 Comparison of two different methods to analyze *Chlamydomonas* RNA-Seq data. √ - The identical pathways in the two methods.
10.1186/s13068-015-0382-0 Comparison of the top hit pathway—Oxidative Phosphorylation Pathway from the two methods. a) Using *Chlamydomonas* genome information to do the alignment and annotation to analyze the RNA-Seq data. b) Using the Bag2D program to construct the *Chlamydomonas* database, and use the self-constructed Cre database for the RNA-Seq data analyses.

## Abbreviations

NGS: next-generation sequencing
Bag2D-workflow: Blast1-annotation1-gene model1-Delete redundant genes-Blast2-annotation2-gene model2
TAGs: triacylglycerols
ACCase: acetyl-CoA carboxylase
DGAT: diacylglycerol acyltransferase
PDAT: phospholipid: diacylglycerol acyltransferase
ME: malic enzyme
FAS: fatty acid synthase
PEPC: phosphoenolpyruvate carboxylase
FDR: false discovery rate
FC: multiple fold change
GO: gene ontology
KEGG: Kyoto encyclopedia of genes and genomes
MIOX: myo-inositol oxygenase
FabG: 3-oxoacyl-[acyl-carrier protein] reductase
*ble*: bleomycin resistance gene
