# Supplementary material for: RNA-Seq transcriptomic analysis with Bag2D software identifies key pathways enhancing lipid yield in a high lipid-producing mutant of the non-model green alga Dunaliella tertiolecta
Source: Biotechnol Biofuels. 2015 Nov 25;8:191. doi: 10.1186/s13068-015-0382-0 (PMC4660794; doi:10.1186/s13068-015-0382-0)
Supplement: Supplementary file 1 — 10.1186/s13068-015-0382-0 Linear regression of Nile red assay and GC–MS measurement. Horizontal error bars show standard deviation for Nile red assay; Vertical error bars show standard deviation for GC–MS assay. The line shows linear regression between the two methods. Strain genotypes 1, 3, and 5 represent culture days 8, 13, and 16 for wild-type strain, respectively; 2, 4, and 6, represent culture days 8, 13, and 16 for D9 mutant strain. All experiments were performed in triplicate. [file 13068_2015_382_MOESM1_ESM.docx]

**Additional file 1 – Correlation between Nile red assays and GC-MS measurements**

Horizontal error bars show standard deviation for Nile red assay; Vertical error bars show standard deviation for GC-MS assay. The linear line shows the correlation between these two methods. Strain genotypes: 1, 3, 5, represent culture day 8, 13, 16 for wild-type strain, respectively; 2, 4, 6, represent culture day 8, 13, 16 for D9 mutant strain. All experiments were performed in triplicate.
