# Supplementary material for: RNA-Seq transcriptomic analysis with Bag2D software identifies key pathways enhancing lipid yield in a high lipid-producing mutant of the non-model green alga Dunaliella tertiolecta
Source: Biotechnol Biofuels. 2015 Nov 25;8:191. doi: 10.1186/s13068-015-0382-0 (PMC4660794; doi:10.1186/s13068-015-0382-0)
Supplement: Supplementary file 4 — 10.1186/s13068-015-0382-0 Run summary of second round of D9 duplicate samples on the Illumina MiSeq platform. [file 13068_2015_382_MOESM4_ESM.docx]

### Additional file 4 - Run summary of second round of D9 duplicate samples on the Illumina MiSeq platform

| **Sample name** | **Sequencing stats and pre-alignment QA/QC of raw data**  **/pre-alignment QA/QC of post-trimming data** | | | | | **Post-alignment QA/QC after alignment** | |
| --- | --- | --- | --- | --- | --- | --- | --- |
|  | Total reads | Avg. read length | Avg. read quality | % N | % GC | Total reads | % Aligned |
| D9-1_S1_L001_R1 | 3,239,491/ **3,239,245** | 133.7/ **133.6** | 37.68/ **37.69** | 0/ **0** | 47.43/ **47.42** | 3,239,245 | 68.94 |
| D9-2_S2_L001_R1 | 3,515,548/ **35,15,368** | 133.58/ **133.5** | 37.74/ **37.75** | 0/ **0** | 46.49/ **46.48** | 35,15,368 | 70.48 |
| WT-1_S3_L001_R1 | 2,223,016/ **2,222,563** | 139.2/ **139.0** | 37.41/ **37.44** | 0/ **0** | 46.13/ **46.12** | 2,222,563 | 52.5 |
| WT-2_S4_L001_R1 | 2,898,342/ **2,898,030** | 134.79/ **134.7** | 37.59/ **37.61** | 0/ **0** | 47.41/ **47.4** | 2,898,030 | 62.8 |
