# Supplementary figures and images for: RNA-Seq transcriptomic analysis with Bag2D software identifies key pathways enhancing lipid yield in a high lipid-producing mutant of the non-model green alga Dunaliella tertiolecta
Source: Biotechnol Biofuels. 2015 Nov 25;8:191. doi: 10.1186/s13068-015-0382-0 (PMC4660794; doi:10.1186/s13068-015-0382-0)

1.
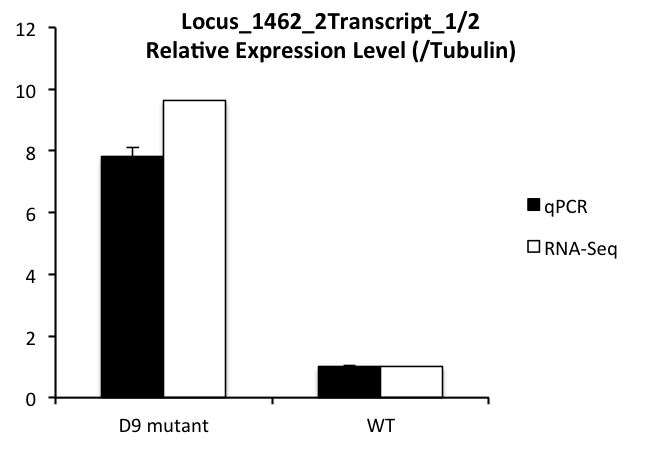

2.
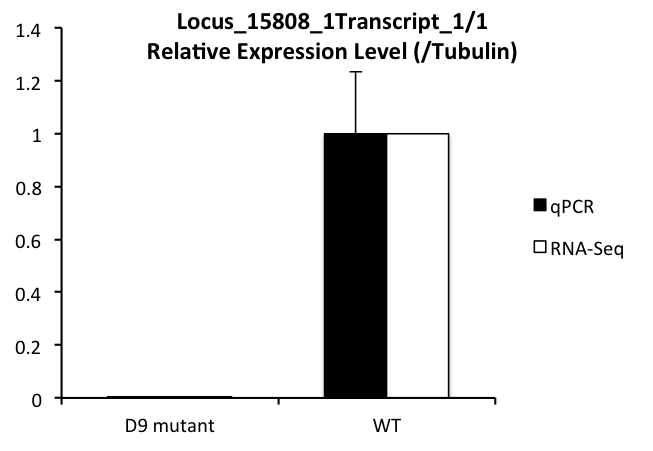


**Additional file 7 -** **Comparison of gene expression profiles from real-time PCR and RNA-Seq**

Supplement: Supplementary file 7 — 10.1186/s13068-015-0382-0 Comparison of gene expression profiles from real-time PCR and RNA-Seq. a) The ratio of Locus_1462_2Transcript_1/2 expression level in D9 compared to wild-type D. tertiolecta is 7.8 from qPCR and 9.6 from RNA-Seq. b) The ratio of Locus_15808_1Transcript_1/1 expression level in D9 compared to wild-type D. tertiolecta is 6.2E − 04 from qPCR and 1.1E − 06 from RNA-Seq. [file 13068_2015_382_MOESM7_ESM.docx]

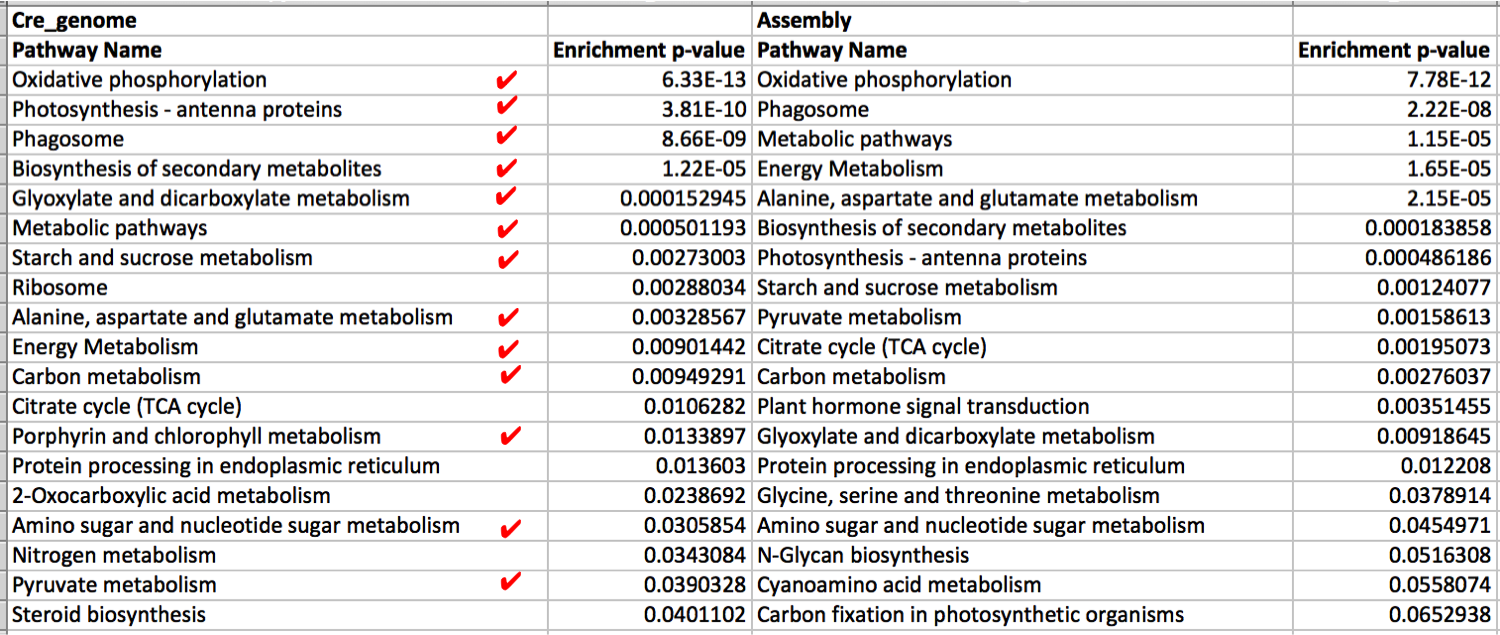


**Additional file 8 - Comparison two different methods to analyze *Chlamydomonas* RNA-Seq data**

Supplement: Supplementary file 8 — 10.1186/s13068-015-0382-0 Comparison of two different methods to analyze Chlamydomonas RNA-Seq data. √ - The identical pathways in the two methods. [file 13068_2015_382_MOESM8_ESM.docx]
