# Supplementary material for: RNA-Seq transcriptomic analysis with Bag2D software identifies key pathways enhancing lipid yield in a high lipid-producing mutant of the non-model green alga Dunaliella tertiolecta
Source: Biotechnol Biofuels. 2015 Nov 25;8:191. doi: 10.1186/s13068-015-0382-0 (PMC4660794; doi:10.1186/s13068-015-0382-0)
Supplement: Supplementary file 9 — 10.1186/s13068-015-0382-0 Comparison of the top hit pathway—Oxidative Phosphorylation Pathway from the two methods. a) Using Chlamydomonas genome information to do the alignment and annotation to analyze the RNA-Seq data. b) Using the Bag2D program to construct the Chlamydomonas database, and use the self-constructed Cre database for the RNA-Seq data analyses. [file 13068_2015_382_MOESM9_ESM.docx]

1.
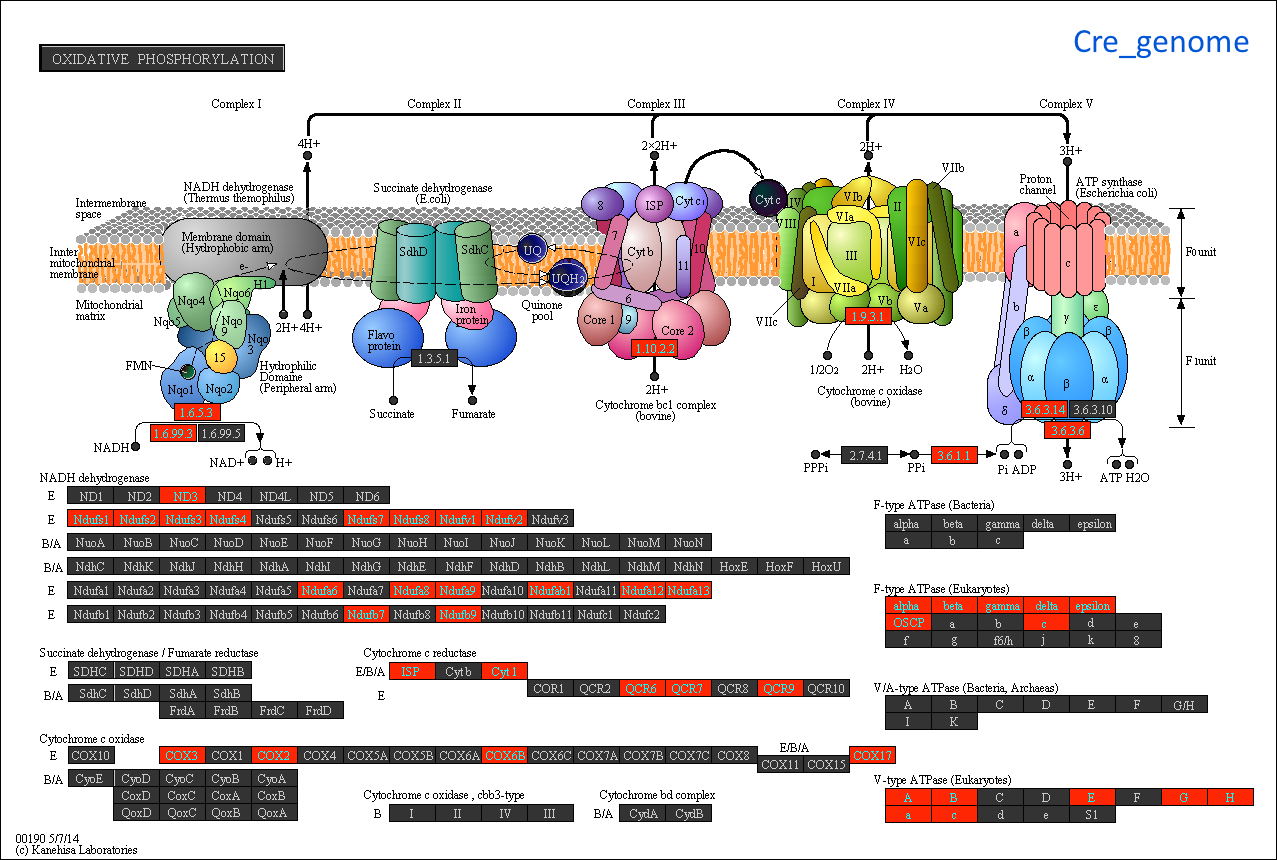

2.
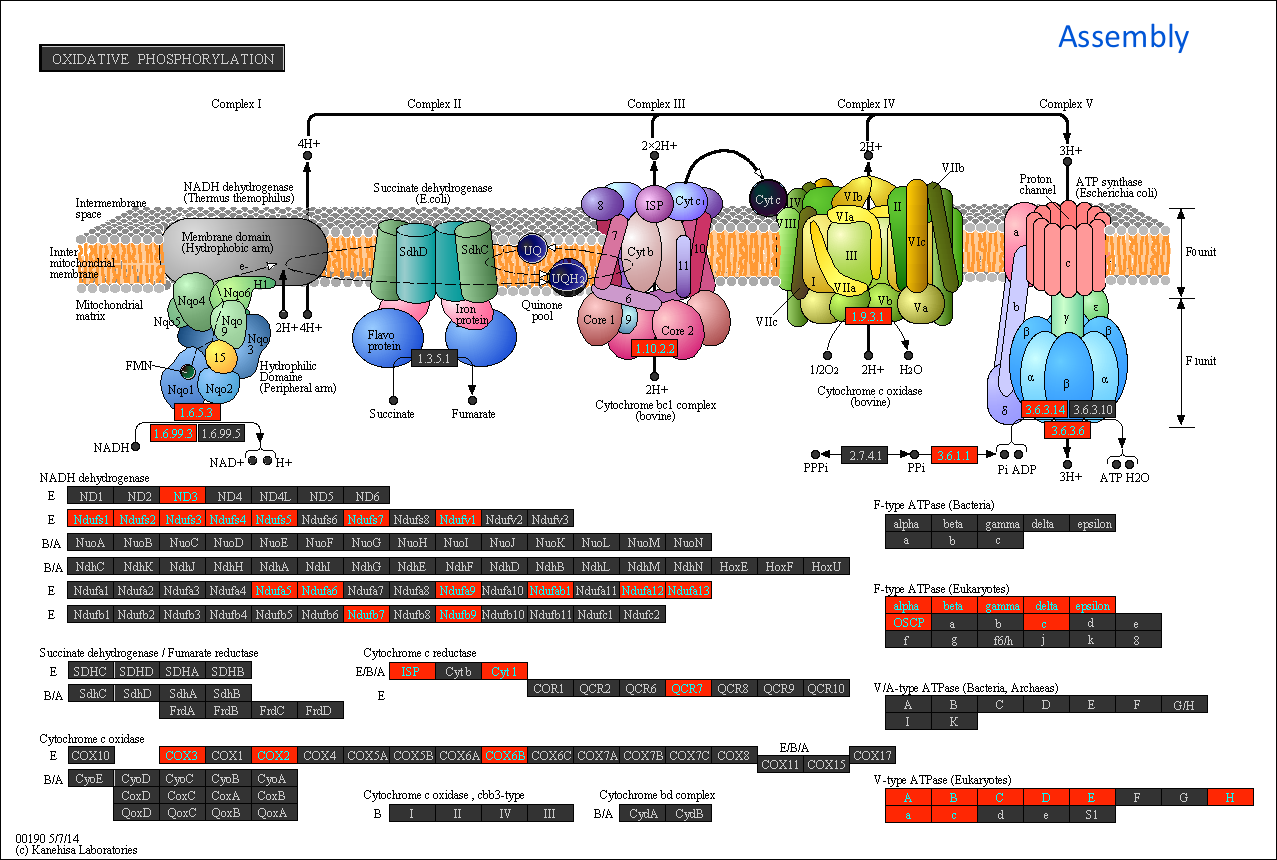


**Additional file 9 - Comparison of the top hit pathway – Oxidative Phosphorylation Pathway from the two methods**
